# Journalism

**Published:** 1867-06

**Authors:** 


### EDITORIAL DEPARTMENT.

*Journalism*—When Scaliger had finished his Lexicon, worn out with his tedious task, and disgusted at the small reputation he could reasonably expect as a reward for such protracted and exhausting labor, he is said to have thrown down his pen and petulantly exclaimed that the dammed in Hell might have their punishment commuted to writing Dictionaries, and not escape a single pang of all their endless torture. The great scholar lived before the days of periodicals, or he would have discovered that there was another literary task still more barren of reputation, still more thankless, still more wearisome than that which provoked his famous outburst of spleen.

The reader who languidly turns over the leaves of his periodical visitant and idly dabbles in a review here, or an editorial there, has little conception of the labor and anxiety which these lines he so lightly skims over have cost the editor. Criticism is easy compared with production, and it is liberally dispensed in proportion to its cheapness. It is amusing to see with what flippancy lucubrations, that were painfully produced with much thought and no little expenditure of midnight oil, are disposed of by the critic.

Now far be it from us to deprecate criticism. Whoever comes before the public is childish if he does not expect it, and incurably silly if he does not profit by it. A wholesome, sound criticism is the life of any literature. Nothing is more disgusting to any man who has a decent regard for his own reputation than indiscriminate and monotonous eulogy. That sort of thing may do for the "Mutual Admiration Society" or half-drunken after dinner speeches, when the orators looking at their friends through the ruddy wine, naturally enough, see them all *couleur de rose.* Every man is then the paragon in his own department, and every speaker finds that to be the proudest moment of his life.

By all means then let us have criticism, but let it avoid flippancy of blame as well as extravagance of praise. Let the censor consider a moment what the task of the editor is. In our own department for example, it is by no means a light one. We have to cater for a great variety of tastes, and to keep the public informed upon very many topics. The advance of several sciences, more or less connected with dentistry, is to be kept pace with. Variety is to be attained. One man is engaged in a particular pursuit and for the time cares for nothing except that. But another has something else in view which he pursues with equal singleness of purpose. The only way, to meet the wants of each then, is to attempt to furnish instruction for all. But even a journalist can claim no exemption from the law of space and time. He might be very glad to cover the whole circle of the science, in a single number, but his paper has a definite limit and his aspirations must be restricted to that. The impatient reader, who does not find what he wants in the first number upon which his eye happens to rest must have a little patience and he may find it in the next.

It is our desire to know the wants of our readers, and our intention to meet them. We therefore ask them to address us letters of inquiry in regard to any special subject they desire to see elucidated. It will afford us pleasure to enlighten them to the full extent of our ability. It is our intention to open a page, or more if necessary, to such correspondents and we invite communications.

